# The School Doctor and Sex Education

**Published:** 1916-12-02

**Authors:** 


### THE SCHOOL DOCTOR.

### The School Doctor and Sex Education.

The recent publication of the Report of the Royal Commission on Venereal Diseases has revived public interest in an educational problem which was exciting much attention before the war. Responsible teachers of all grades have felt for some time that an education which ignores the question of sex, and makes no serious attempt to train the young for some of the gravest responsibilities in life, is indefensible in theory and disastrous in practice. The Report of the Royal Commission has given point to this contention by exposing the full measure of the "perils of ignorance," and furthermore by laying great emphasis on the value of educational measures as a prophylaxis against the ravages of venereal disease. "It is, in our opinion, absolutely necessary that the public should have fuller knowledge of the grave evils which exist among us, and of their effect upon the national life, present and future. At the same time, we believe that instructions and warning should be given to the young of the moral and physical dangers which may imperil them."

### Sense and Sensibility.

The question is, of course, a wider one even than is here indicated, and must be approached from a broader standpoint than that of prevention of disease. Experience has shown that little success is to be hoped from presenting the dangers of immorality to young people as an isolated fact, even when "moral principles and spiritual considerations" are included as the Commissioners suggest. Unless it is linked up with instruction on general hygiene and physiology on the one hand, and lessons on temperance in respect to other appetites (for example, the use of alcohol) on the other, sex teaching inevitably gets out of focus and degenerates into sensationalism or sentimentality, unwholesome for teacher and pupils. What is needed is a coherent national scheme for sex education, co-ordinated at all points with existing teaching of hygiene, biology, and morals, and carefully planned by persons who know something of sex, its physiology and pathology, and something of education. This can only be achieved if education authorities can be persuaded to take the matter in hand, with the co-operation of their medical officers and any other experts in hygiene on their staffs. At present it is the happy hunting ground of cranks and faddists of every description.

### The Responsibility of the Parent.

In constructing a scheme of sex education it has to be recognised that much of the information which it is desired to convey to the young cannot be imparted publicly, more especially in the elementary school. The parent is generally acknowledged to be the proper person to initiate the child into the intimate mysteries of life. The first move in the campaign is, therefore, an indirect one, and consists in the instruction of adults, not only parents, although they are the most important factors in the situation, but also teachers and social workers and others charged with the training of children and young people. The instruction given varies with the type of audience, but would include a brief account of the physiology of reproduction, the social and physical consequences of immorality, the best methods of training children in good habits and self-control. Hints on suitable ways of imparting information to little ones about the origin of life are of great practical value. It is found that teachers attend such courses very readily, but parents, especially those whose children need help most, are by no means easy to approach. If anything really widespread and effective is to be done in this, the most fruitful form of sex education, the schools will have to be used as a focus for attracting the parents. A group of mothers, assembled to meet a school doctor whose personality and advice they have learnt to value, constitutes an ideal type of audience.

It is not right, however, to ignore the claims of the child whose parents for one reason or another will take no share in enlightening it. The Royal Commissioners, therefore, advocate lectures to young people in evening continuation schools, factories, and workshops. Although the most direct method of disseminating knowledge, this is in many respects the least satisfactory. The natural sensitiveness of adolescence constitutes a very real difficulty to the most tactful lecturer, and where the peculiar prudishness of uneducated persons is superadded, the chances of doing more harm than good are considerable. Excellent results have occasionally been obtained where the lectures formed part of a course, or were judiciously led up to by talks on simple health topics.

### Foundation Laying at the Schools.

The success of all attempts to instruct adolescents clearly depends very largely on the foundation that has been laid in the elementary school. The fact that children leave at fourteen, when admittedly "their immaturity makes detailed instruction on every ground most undesirable," has caused the schools' important share in sex training to be overlooked, even by the teachers and doctors who should know better. For example, no amount of lectures or studying pamphlets can be expected to turn the average parent into an efficient teacher of biology, it is in school that his children should get a knowledge of the basic principles of the transmission of life. As Miss Norah March has shown, it is possible without giving offence to carry class teaching to a point where a few simple words from the parent can link up reproduction in plants with the same phenomena in animals and man. The teaching of hygiene affords equally valuable opportunities in skilful hands. It has improved enormously of recent years, and school medical officers are able to testify that many children now leave with a sound, though necessarily elementary, knowledge of their bodily functions and of the laws of healthy living. Furthermore, the cult of personal cleanliness as carried out under an enthusiastic staff constitutes a very efficient training in self-control and respect for the body, principles which are the basis of all sane sex instruction. Children so trained have a real defence against temptations, and are well prepared for detailed and specialised teaching given either in private talks with teachers or parents or in class in after years. By encouraging their authorities to raise the often deplorably low level of hygiene teaching in the worst schools to the heights it can attain in the best, school doctors can perform a most valuable service to the community.

